# mSphere of Influence: Venturing outside the biology canon with sex and gender

**DOI:** 10.1128/msphere.00594-24

**Published:** 2024-11-06

**Authors:** Victoria Prieto-Echagüe

**Affiliations:** 1 Laboratory of Human Molecular Genetics, Institut Pasteur de Montevideo, Montevideo, Uruguay; Institut Pasteur de Montevideo, Montevideo, Uruguay

**Keywords:** sex as a biological variable (SABV), gender-inclusive research, precision medicine

## Abstract

Victoria Prieto-Echagüe works in the field of signaling by primary cilia, adipogenesis, and obesity. In this mShpere of Influence article, she reflects on how gender studies, feminism, and societal movements such as #metoo may inform all areas of biomedical and health research. She describes how they inspired her to incorporate sex as a biological variable (SABV) principle to her research exploring sex-specific mechanisms in obesity and metabolic diseases and argues that incorporating SABV is crucial for advancing precision medicine and addressing healthcare inequities.

## COMMENTARY

In biology and healthcare, the male body has historically been the standard. As a result, the female body has been understudied, leading to gaps in the knowledge about women’s health. Thus, female-specific pathologies or even a sex-specific presentation of a known pathology have gone unrecognized or treated as deviations from the norm. This neglect in research parallels broader societal issues, such as contemporary movements like #MeToo, massive demonstrations like “Ni una menos” in Argentina, and the International Women’s Strike in 2017. These movements sparked conversations about systemic abuses, domestic violence, and gender harassment in society including research and academia.

As a woman scientist, I was deeply moved by the data revealing entrenched yet long overlooked inequalities. This prompted me to engage in collective work generating original data and raising awareness about the power dynamics that perpetuate these inequalities often described as the “glass ceiling.” I advocated for gender equality in research and academia, to expose the barriers faced not only by women but also by non-hegemonic groups historically marginalized due to ethnicity, LGBTQ+ identity, or geographical location.

Feminist and gender studies scholars paved the way for mainstreaming the gender perspective. Realizing the importance of a social perspective, I pursued formal education in social studies. This helped me appreciate the distinction between sex and gender, terms often confused or ignored in scientific research. Sex refers to the biological characteristics ranging from chromosomal complement to hormone metabolism and may be conceived as a continuum (1). In contrast, gender refers to the societal norms that regulate behaviors, expectations, and power dynamics that are built around biological differences. Kimberlé Crenshaw’s concept of intersectionality, deeply influenced my approach, as it explains how a given individual experiences multiple axes of privilege or oppression that intersect and combine to create a much greater harm (2). I realized that adopting an intersectional approach in both my research and advocacy, was essential to create a more powerful and just frame of thought.

Turning this feminist lens to my background in biology, I noticed another oversight. Even as precision medicine becomes a trend in translational research, there is an unexplained failure to account for sex as a biological variable (SABV), thereby creating gaps of knowledge and access to appropriate healthcare for everyone. These gaps are in part due to the lack of research, with more funds allocated to research diseases that are more prevalent in men. Prime examples of this are migraines, anorexia, or endometriosis that affect primarily females, and their research is seriously underfunded in comparison to the burden they represent (3). Work by Londa Schiebinger, groundbreaking and paradigm-shifting work emphasizes that almost any research topic can be interrogated for sex and gender implications (4). Similarly, Cara Tannembaum and other trailblazing scientists and policy makers argued for including both sex and gender as variables in biomedical research (5), leading to policy changes that incorporated SABV requirements in the United States, Canada, and Europe along the research process from grant applications, preclinical and clinical studies design and publishing (6, 7). The importance of this shift is crucial. For example, several drugs, notoriously including the sleep medicine Zolpidem, were withdrawn or dose-adjusted after it was discovered that they affected men and women differently. It is vital to realize that the failure to include female individuals in preclinical and clinical studies may lead to extremely costly errors in terms of public health and resources. Alondra Nelson’s speech where she stated that “Science at its core is a social phenomenon” was another turning point for me (8). Her perspective reinforced that incorporating sex and gender into research is not just ethical but essential for scientific rigor.

Since then, I find more inspiration in articles reporting important discoveries by incorporating research designs hitherto overlooked. Cattaneo et al. provided support for considering the cellular sex in culture conditions. Endothelial cells from male or female umbilical cords were cultured and then subjected to a source of stress, such as serum starvation. The proteins secreted by these cells to the media are a sexual dimorphic phenotype, further suggesting that a male fetus might respond differently from a female fetus to the same stimulus (9). Another striking example comes from a study in cancer whereby considering sex as a continuous variable in a cancer transcriptomic analysis showed that sex-specific mechanisms may underlie the pathogenesis of cancer, taking precision treatments a step further (10).

In my own research, this feminist perspective was a powerful inspiration fundamentally changing my research on signaling during adipogenesis and adipose tissue characterization in obesity. I became interested in studying whether the sex of the primary cell cultures used for adipogenesis experiments matters. In addition, I have moved onto *in vivo* models to investigate metabolic phenotypes where all the features of biological sex are at play. By following up in the observation that a particular mutation causes a much greater weight gain in female mice than in male mice, I have been able to expand on previous characterizations of the obesity phenotype linked to a ciliopathy. In practice, I have taken advantage of statistical tools that recommend using a factorial design followed by a two-way ANOVA test to improve the statistical power of experiments without necessarily doubling the number of individuals and therefore the costs in a study.

Looking to the future, SABV inclusion must be consolidated and extended from artificial intelligence to infectious diseases, including, cancer, heart, and metabolic disease (4). One of the next great challenges in precision medicine research is including and making gender an operational variable in studies and data processing (11). As I reflect on what has most impacted my approach to biological science, I proudly realize that the inspiration for my current scientific focus has been shaped and improved by feminist thought and social sciences. As bell hooks famously said, “Feminism is for everybody,” and this perspective has enriched my understanding of biology and has inspired me to approach my work with a broader, more inclusive perspective, hopefully leading to a more equitable, impactful science.
